# Identification and Ligand-Binding Mechanism Analysis of a C4-Dicarboxylate Chemoreceptor from *Agrobacterium tumefaciens*

**DOI:** 10.3390/biom16101405

**Published:** 2026-09-27

**Authors:** Jinjing Liu, Nan Xu, Hao Wang, Minliang Guo

**Affiliations:** College of Bioscience and Biotechnology, Yangzhou University, Yangzhou 225009, China; dx120210194@stu.yzu.edu.cn (J.L.); nanxu@yzu.edu.cn (N.X.); wanghao@yzu.edu.cn (H.W.)

**Keywords:** *Agrobacterium tumefaciens*, chemotaxis, methyl-accepting chemotaxis protein, ligand recognition, amino acid substitution

## Abstract

Methyl-accepting chemotaxis proteins (MCPs, or chemoreceptors) function as sensors and signal transmitters of the chemotaxis signaling system. As a soil-borne facultative phytopathogen, *Agrobacterium tumefaciens* (or *fabrum*) C58 encodes 20 MCPs, but only seven have been experimentally verified. Here, we characterized Atu0646, predicted as a transmembrane chemoreceptor with an sCache_2-type periplasmic ligand-binding domain (LBD_0646_). Differential scanning fluorimetry (DSF) and isothermal titration calorimetry (ITC) results confirmed LBD_0646_ directly binds L-malate and succinate. Gene deletion assays validated Atu0646 as the chemoreceptor for these two ligands in *A. tumefaciens* C58. The chemotactic attraction of *A. tumefaciens* toward two plant-derived C4-dicarboxylates provides reasonable support for the critical role of chemotaxis in rhizosphere colonization and susceptible wound site localization of this phytopathogen. AlphaFold structural modeling, molecular docking, and site-directed mutagenesis identified key binding residues: five substitutions (Y90A, H103A, K155A, R125Y, Y174K) resulted in loss of function due to the alteration of its LBD structure, two (R108Y, N151Y) attenuated it, and one (S119Y) enhanced its binding capacity to its cognate ligand, while also broadening its ligand spectrum to recognize Asp. Ligand-binding mechanism analysis of Atu0646 improves our understanding of the structure–function relationship of chemoreceptors and provides a feasible strategy for the rational protein engineering of chemoreceptors.

## 1. Introduction

Chemotaxis is defined as a behavioral process in which motile bacteria and archaea sense and track the gradients of chemoattractants and chemorepellents in their surroundings and subsequently navigate toward favorable environments or away from harmful chemicals [1]. This mechanism plays a critical role in bacterial ecology and is essential for survival, particularly by enabling microorganisms to gain a competitive advantage in colonizing inhospitable and nutrient-depleted environments [2,3]. Chemotaxis is primarily driven by the acquisition of nutritional substances essential for growth, as evidenced by observed migratory responses toward sugars, amino acids, organic acids, and inorganic phosphate [3,4]. In addition, chemotaxis has been documented in response to other chemicals such as neurotransmitters, plant hormones, and quorum signaling molecules, as well as certain physical signals [5,6,7,8,9].

The chemotaxis signaling pathway is a non-canonical two-component signaling system. The system comprises a ternary core complex consisting of methyl-accepting chemotaxis proteins (MCPs, now more often called as chemoreceptor), the coupling protein CheW, and the histidine kinase CheA, together with a phosphorylatable response regulator protein, CheY [10,11,12,13,14]. Chemotaxis is primarily mediated by MCPs. Most MCPs are transmembrane chemoreceptors. A typical MCP exists as a homodimer formed by two identical subunits, and each subunit contains three structural modules: a periplasmic ligand-binding domain (LBD), one or more transmembrane helices (TMs), and an extended helical cytoplasmic signaling domain (CSD) [10,11,12]. The LBD acts as the signal perception module. From the membrane-proximal region to the membrane-distal tip, the CSD can be sequentially divided into four regions (or subdomains): the HAMP (existing in histidine kinases, adenylate cyclases, methyl-accepting proteins and phosphatases) domain, methylation helix bundle (MHB) region, flexible bundle region and kinase control region (or protein interaction region) [12,13]. Glutamyl residues within the MHB are capable of methylation and demethylation. The designation of methyl-accepting chemotaxis protein (MCP) derives from its capability to receive methyl groups. When a chemoeffector (chemoattractant or chemorepellent) binds to the LBD of a transmembrane MCP, the MCP’s CSD transduces the signal to the histidine kinase CheA via the coupling protein CheW, thereby modulating CheA activity. The dimeric histidine kinase CheA undergoes trans-autophosphorylation, where one CheA subunit phosphorylates the other, and subsequently transfers the phosphate group to CheY [11,12]. Phosphorylated CheY (CheY-P) regulates the rotation of flagellar motor by binding to FliM and FliN, two components of the basal motor switch complex, ultimately triggering the chemotactic response [14]. MCP methylation levels govern their capability to modulate CheA activity, adjusting MCPs’ sensitivity to the ligand to adapt to varying ligand concentrations [14,15].

To sense diverse chemicals, bacteria with chemotactic behavior have evolved a large number of chemoreceptors. Analysis of sequenced genomes has shown that each bacterial genome carries an average of up to 14 chemoreceptor genes [16]. Bacterial lifestyle has also been shown to influence the quantity of chemoreceptors. For instance, bacterial species capable of colonizing diverse and fluctuating habitats possess roughly five times as many chemoreceptors as those restricted to a single, defined ecological niche [16,17,18].

Chemoreceptors differ in cellular localization, topology, sensing mode, and LBD type. However, the substantial divergence within the chemoreceptor family lies mainly at the levels of protein topology and LBD type, whereas the CSD is conserved across all chemoreceptors [18,19,20]. Based on computational identification of LBDs and sequence-derived LBD classification, up to one hundred distinct types of sensor domains were identified in the predicted LBDs of MCP sequences, which are defined by the conserved methyl-accepting subdomain within the signaling domain. Yet only a few LBD types are prevalent: 33% Cache (found in calcium channels and chemotaxis receptors), 31% 4HB (a long dimeric four-helix bundle formed by two symmetric antiparallel coiled coils), 19% PAS (found in Per-Arnt-Sim proteins), 3% CZB (chemoreceptor zinc-binding domain), 3% GAF (found in cGMP-specific phosphodiesterases, adenylyl cyclases, and FhlA), and 3% protoglobin [19]. These abundant LBD types were further classified into several subclasses and sub-subclasses. For instance, PAS has been classified into 17 families [20,21]; the Cache type comprises double Cache (dCache) and single Cache (sCache); dCache includes dCache_1, dCache_2, dCache_3, Cache_3-Cache_2, and so on; sCache includes sCache_2, sCache_3_2, sCache_3_3, CHASE (cyclase/histidine kinase-associated sensory extracellular fold), and so on [19,22]. However, it is very often that the same LBD type can recognize different ligands and some ligands can bind to structurally different LBDs. The absence of a clear structure–function relationship for LBDs makes it difficult to accurately predict chemoreceptor functions by simply extrapolating from homologous proteins that have been experimentally characterized [12,19,23]. In addition, certain chemoreceptors—particularly those lacking LBDs—are able to bind their ligands indirectly via interactions with partner proteins [10,12]. Therefore, functions of each chemoreceptor are required to be identified by experimental approaches.

For pathogenic microorganisms, MCPs serve a critical dual role by recognizing chemical signals and mediating pathogen–host interactions [7,9,24]. Particularly, plant-associated bacteria possess a larger number of MCPs, indicating that the chemosensory system is an important requisite for plant–bacterium interactions. In fact, chemotactic behavior has been shown to play a critical role in the establishment of plant–microbe interactions [9,17]. Characterization of chemoreceptors is essential for studying plant–microbe interactions and bacterial virulence. *Agrobacterium tumefaciens* is a soil-borne phytopathogen capable of infecting dicotyledonous plants under natural conditions [25,26]. Chemotaxis plays a vital role in host recognition and locating infection sites in host plants for *A. tumefaciens* [15]. As a facultative plant pathogen, this bacterium survives in diverse habitats ranging from bulk soil and rhizosphere to plant-associated niches. Its adaptable lifestyle has led to the evolution of 20 chemoreceptors. The LBDs of 20 chemoreceptors from *A. tumefaciens* C58 strain fall into 6 LBD types: dCache_1, sCache_2, Cache_3_2, CHASE3, PAS, and protoglobin [12,15,27].

Among the 20 chemoreceptors in *A. tumefaciens* C58, only seven have been experimentally characterized [12]. Loss of the chemoreceptor encoded by *atu0514* impairs chemotactic attraction of *A. tumefaciens* toward acetosyringone (AS), sucrose, valine and citrate; however, any direct or indirect interactions between the Atu0514 chemoreceptor and these four compounds remain uncharacterized [28]. Both chemoreceptor–ligand interaction assays and phenotypic analyses of bacterial mutants confirm that the *atu0526*-encoded chemoreceptor is the sole formate-sensing chemoreceptor in *A. tumefaciens* [29]. Chemotactic responses of the *atu1912*-encoded chemoreceptor to pyruvate and propionate were validated via both chemoreceptor–ligand interaction assays and mutant phenotypic testing [30]. Differential scanning fluorimetry (DSF) revealed that the LBD of the *atu2173*-encoded chemoreceptor interacts with all 17 proteinogenic amino acids excluding Gly, Glu and Asp. Chemotactic attraction toward these 17 amino acids mediated by the Atu2173 chemoreceptor was further verified using an *atu2173*-deficient *A. tumefaciens* mutant strain [31]. Deletion of the *atu1027* gene not only eliminates aerotaxis in *A. tumefaciens* but also weakens chemotaxis of *A. tumefaciens* toward multiple carbon sources [32]. Chemosensing of the *atu0373*-encoded chemoreceptor for choline, acetylcholine, betaine and L-carnitine have also been validated via chemoreceptor–ligand interaction and mutant phenotypic assays [33]. The function of the *atu0872*-encoded chemoreceptor was confirmed only via mutant phenotypic analysis; it is a phenolic acid-sensing chemoreceptor. Deletion of *atu0872* not only impairs chemotaxis of *A. tumefaciens* toward multiple phenolic acids, but also attenuates its pathogenicity [34]. Functional characterization of additional *A. tumefaciens* chemoreceptors will deepen our understanding of its niche and host adaptation, as well as its virulence. Here, we functionally characterize the chemoreceptor encoded by *atu0646* (hereafter referred to as Atu0646).

Sequence analysis of Atu0646 reveals that its LBD is an extracellular sensor domain and belongs to the sCache_2 type [12]. Cache-type domains are the most abundant extracellular LBD family in prokaryotes and are known to recognize a broad diversity of ligands. However, to date, only a few sCache_2-containing LBDs have known ligands although Cache domains are among the best-characterized chemoreceptor sensor domains [22]. Known ligands for sCache_2 type LBDs include organic acids (e.g., L-lactate, acetate, acetoacetate, glycolate, malonate, pyruvate, L-malate, and citramalate), substituted carboxylic acids (e.g., bromosuccinate), and amides (e.g., urea, hydroxyurea, acetamide, and formamide) [22,23]. The high sequence variation among sCache_2 type LBDs and the diversity of their known ligands prohibit reliable prediction of Atu0646 function. Therefore, we use multiple experimental approaches, including differential scanning fluorimetry (DSF), isothermal titration calorimetry (ITC), and site-directed mutagenesis, to identify the ligands of Atu0646 and further elucidate the mechanism of ligand binding.

## 2. Materials and Methods

### 2.1. Bacterial Strains, Plasmids, and Primers

The strains, plasmids and primers used are listed in Supplementary Materials (Tables S1 and S2). *Escherichia coli* was cultured in lysogeny broth (LB) agar (1.5%, *w*/*v*) or liquid medium at 37 °C [35,36]. *A. tumefaciens* was cultured in MG/L or AB-sucrose agar (1.5%, *w*/*v*) or liquid medium at 28 °C [37,38]. During liquid cultivation, the shaking speed was 220 rpm for *E. coli* and 200 rpm for *A. tumefaciens*. When cultured under selective conditions, the final concentration of kanamycin was 50 µg/mL for *E. coli* and 100 µg/mL for *A. tumefaciens* [37,38].

### 2.2. DNA Preparation

The standard molecular protocols were used to amplify the gene, purify the DNA, and construct the plasmid [38]. *A. tumefaciens* gene sequences refer to the published genome sequence (genome accession number: AE007869.2). Plasmid DNA was extracted and purified with TIANprep Mini Plasmids Kit (Tiangen Biotech Corporation, Beijing, China). PCR products and DNA fragments were purified from the gel by using MolPure^®^ Fast GelExtraction Kit (Yeasen Biotechnology Corporation, Shanghai, China) [39]. Plasmids were transformed into *E. coli* DH5α competent cells through heat-shocking at 42 °C [36] and *A. tumefaciens* through electroporation [38].

### 2.3. Construction of Mutant and Complementary Strains

The precise deletion mutant of the *atu0646* gene in *A. tumefaciens* was constructed following the previously described gene replacement procedure [38,40]. The gene deletion strategy mainly utilized the pEX18Km plasmid, which harbors a kanamycin resistance gene serving as a positive selection marker and the suicide gene *sacB* for counter-selection. A 500-bp fragment upstream of the *atu0646* start codon was fused to another 500-bp fragment downstream of its stop codon. The fused fragment was ligated into the pEX18Km plasmid, and the resulting recombinant plasmid was introduced into *A. tumefaciens* via electroporation. Single crossover transformants were screened on kanamycin-containing medium by virtue of the plasmid-encoded kanamycin resistance. After being verified by PCR, single crossover transformants underwent a second round of counter-selection using 5% (*w*/*v*) sucrose. Target gene-deletion mutants were screened from double crossover transformants via PCR and further verified by DNA sequencing.

The complementary strain was constructed by introducing a plasmid carrying *atu0646* gene with its native promoter (a fragment located approximately 500-bp upstream of the start codon) into the gene deletion mutant [37,39]. The entire complementary DNA fragment was amplified by PCR and cloned into the self-replicating pCB301 plasmid. The resulting construct was subsequently transformed into the gene deletion mutant. The correct complementary strain was ultimately verified through confirmation assays.

The single-residue mutation was carried out as described previously [41]. The circular complementation plasmid was used as a template for PCR amplification with a pair of perfectly matched primers designed to introduce a single amino acid mutation. The resulting PCR product was a full-length, non-methylated plasmid. Since the original template plasmid was methylated due to its extraction from *E. coli* DH5α, the template could be efficiently digested with *Dpn* I endonuclease. As a result, only the newly amplified mutated plasmid remained in the final product.

### 2.4. Chemotaxis Assay

The chemotactic response to a single substance was validated using the capillary assay as described previously [37]. *A. tumefaciens* cells were grown to the logarithmic growth phase in AB-sucrose liquid medium, harvested by centrifugation at 4000 rpm for 10 min at room temperature (25 °C), washed twice with chemotaxis buffer (0.1 mmol/L EDTA and 10 mmol/L KH_2_PO_4_, pH 7.0), and finally suspended in fresh chemotaxis buffer to an OD_600nm_ of 0.1. Each 300 μL aliquot of suspension was designated as a bacterial pond. Capillaries were filled with the single attractant at desired concentrations (dissolved in chemotaxis buffer) and then sealed at one end. The open end of the capillary tube was inserted into the bacterial pond and incubated for 1 h at room temperature; then, the solution in the capillary tube was expelled and completely transferred into 1 mL of 0.85% saline. Each 10 μL aliquot of the dilution was plated on LB agar plates and incubated at 28 °C for 36 h. The colonies in the plate were counted and represented the number of the cells attracted to the capillary tube.

### 2.5. Protein Expression and Purification

The periplasmic domain of Atu0646 (from 27th residue to 189th residue, LBD_0646_) was expressed using pET30a with a C-terminal 6-histidine tag. Recombinant proteins were expressed in *E. coli* BL21 (DE3). When *E. coli* cells harboring the recombinant pET30a plasmid had been grown at 37 °C to an OD_600nm_ of 0.4 to 0.6, and IPTG (isopropylthio-β-D-galactoside) was then added to the cultures at a final concentration of 0.7 mmol/L to induce the expression of the fusion proteins. After a 4-h induction culture with shaking at 150 rpm and 25 °C, the induced bacterial culture was harvested by centrifugation at 8000 rpm for 6 min, and washed twice with PBS buffer (10 mmol/L, phosphate-buffered saline, pH 7.4). The entire collection procedure was maintained at 4 °C. The cell suspension was sonicated until nearly transparent and then centrifuged at 12,000 rpm for 30 min to remove cellular debris. The resulting supernatant (crude protein extract) was incubated with 2 mL of ProteinIos Ni-IDA Resins (Transgen Biotech, Beijing, China) for 1 h with gentle rotation. The contaminating proteins were subsequently eluted using washing buffers with increasing imidazole concentrations. Finally, the His-tagged target protein was eluted with a 250 mmol/L imidazole solution, and the imidazole was subsequently removed via dialysis [29,37].

### 2.6. Differential Scanning Fluorimetry (DSF)

The binding of a ligand to the protein typically resulted in an alteration of the protein’s thermal stability. DSF was employed to assess the interaction between the ligand and the protein by measuring its thermal stability parameters [42,43]. Proteins at specified concentrations were thoroughly mixed with potential ligand solution (final concentrations of protein and ligand were 10 μmol/L and 1 mmol/L) and SYPRO Orange (5000 × dilution, Thermo Fisher Scientific, Waltham, MA, USA). The sample mixtures were transferred into 8-tube strips, with a volume of 25 µL per well. Protein buffer (50 mmol/L Tris-HCl, 300 mmol/L NaCl, pH 8.0) served as the blank control. All sample preparations were performed at 4 °C. The mixtures were then heated from 15 °C to 95 °C using a CFX Connect Real-Time PCR Detection System (Bio-Rad Laboratories, Hercules, CA, USA)at a scanning rate of 0.5 °C every 30 s. Unfolding curves of proteins were monitored by detecting changes in fluorescent intensity. Melting temperatures were determined from the derivative values of fluorescence data using Bio-Rad CFX Manager 3.1 software (Bio-Rad Laboratories, Hercules, CA, USA) [42,43].

### 2.7. Isothermal Titration Calorimetry (ITC)

Titrations of LBD_0646_ with a ligand were carried out on MicroCal iTC_200_ (Malvern Panalytical, Malvern, UK). In brief, the purified protein sample was adjusted to a concentration of 50 μmol/L with buffer solution (50 mmol/L Tris-HCl, 300 mmol/L NaCl, pH 7.25). A volume of 200 μL was aspirated using the loading needle and slowly injected into the thermostatted sample cell. The ligand solution was proportionally adjusted to the specified concentration with the same buffer solution for preparing the protein sample and loaded into the syringe. Maintaining a constant temperature of 25 °C, the syringe continuously titrated the ligand into the sample cell at a defined rate. The syringe also provided mixing functionality throughout the reaction period. The instrument monitored the thermal changes in the sample cell and maintained equilibrium with the reference cell, resulting in detectable endothermic or exothermic peaks. The data were analyzed using the Origin 7.0 software package (OriginLab Corporation, Northampton, MA, USA).

### 2.8. Molecular Docking Analysis

The molecular docking analysis was performed using AutoDock Vina v1.2.3 (The Scripps Research Institute, La Jolla, CA, USA) [44]. The three-dimensional structure of the LBD_0646_ protein was predicted and analyzed using the SWISS-MODEL (https://swissmodel.expasy.org/interactive; accessed on 22 March 2026) and AlphaFold servers (https://alphafold.ebi.ac.uk/; accessed on 22 March 2026). The three-dimensional structures of small molecule compounds were obtained from the PubChem database (https://pubchem.ncbi.nlm.nih.gov/; accessed on 17 March 2026). The higher-ranking docking poses were selected for further analysis. Their molecular interactions were subsequently visualized and analyzed usingPyMOL 2.6 (Schrödinger, New York, NY, USA; for three-dimensional representation).

### 2.9. Statistical Analysis

All experimental data were derived from at least three independent replicates and were presented as mean ± standard deviation (SD). Statistical significance was determined using unpaired Student’s *t*-test for comparisons between two groups. A *p*-value of less than 0.05 was considered statistically significant.

## 3. Results

### 3.1. LBD of Atu0646 Binds to Two C4-Dicarboxylates

In the *A. tumefaciens* genome database, *atu0646* was annotated as the *mcpA* gene. Sequence analysis of the *atu0646*-encoded protein shows that it is a typical methyl-accepting chemotaxis protein (MCP, or chemoreceptor) comprising two transmembrane helices, a periplasmic sCache_2 type LBD, two HAMP domains, and a highly conserved MA (methyl-accepting) domain that defines MCP family members (Figure 1A). It is notoriously difficult to purify transmembrane proteins in their native conformations. Fortunately, for most chemoreceptors with an extracellular LBD, the LBD can be recombinantly expressed as a standalone protein while retaining native ligand-binding activity [42]. To identify the ligand of the transmembrane chemoreceptor Atu0646, we aimed to express and purify its LBD as a standalone recombinant protein, and further confirm its structural stability. Based on sequence analysis, residues 27–189 of Atu0646 were defined as the LBD, and a 6 × His tag was fused to its N-terminus for purification. The recombinant Atu0646 LBD (hereafter LBD_0646_) was purified. Purified LBD_0646_ exhibited a typical thermal denaturation profile with a melting temperature (T_m_) of 39 ± 0.5 °C in DSF assays, confirming stable folding in solution (Figure 1B).

Previous studies demonstrated that organic acids are the major known ligand class for sCache domain [22]. We screened 54 chemicals bearing carboxyl groups to identify potential ligands of LBD_0646_ using DSF. The binding of specific ligands resulted in increased protein stability, which was manifested as a rise in the melting temperature (T_m_). A compound is considered to bind a protein if it increases the protein’s T_m_ by >2 °C; conversely, no binding is indicated if T_m_ remains unchanged upon compound addition. Among the 54 screened chemicals, only L-malate and succinate increased the T_m_ of LBD_0646_ by >2 °C (ΔT_m_ > 2 °C) (Figure 2A). Concentration gradients of both chemicals were tested to validate binding affinity, with the expectation that T_m_ would rise with increasing ligand occupancy [45]. Consistent with this expectation, higher ligand concentrations produced dose-dependent increases in ΔT_m_ (Supplementary Figure S1). We further quantified the binding affinities. The dissociation constant (K_d_) of the LBD-ligand complex was used to quantify the affinity and was readily determined using ITC. As shown in Figure 2B, both ligands bound directly to LBD_0646_, with K_d_ values of 11.1 ± 0.04 μmol/L and 198 ± 16.1 μmol/L, respectively. Thus, L-malate and succinate were preliminarily identified as the cognate ligands of the Atu0646 chemoreceptor.

### 3.2. Atu0646 Is a Chemoreceptor for L-Malate and Succinate in A. tumefaciens C58

To verify whether Atu0646 functions as the chemoreceptor mediating chemotaxis of *A. tumefaciens* toward L-malate and succinate in vivo, two derived strains of *A. tumefaciens* were constructed: an *atu0646*-deletion mutant (Δ*atu0646*) and its complemented strain (Δ*atu0646*-C). Chemotaxis of three *A. tumefaciens* strains, wild-type (C58), Δ*atu0646* and Δ*atu0646*-C, toward L-malate and succinate was quantified using the capillary assay. As shown in Figure 3, both C58 and Δ*atu0646*-C exhibited robust positive chemotaxis toward both compounds, whereas Δ*atu0646* displayed no detectable chemotaxis toward either ligand. Furthermore, wild-type strain C58 showed a significant chemotactic attraction to both L-malate and succinate at concentrations ranging from approximately 0.1 to 60 mmol/L. These results confirmed that Atu0646 is the chemoreceptor responsible for sensing L-malate and succinate in *A. tumefaciens* C58.

**Figure 2:**
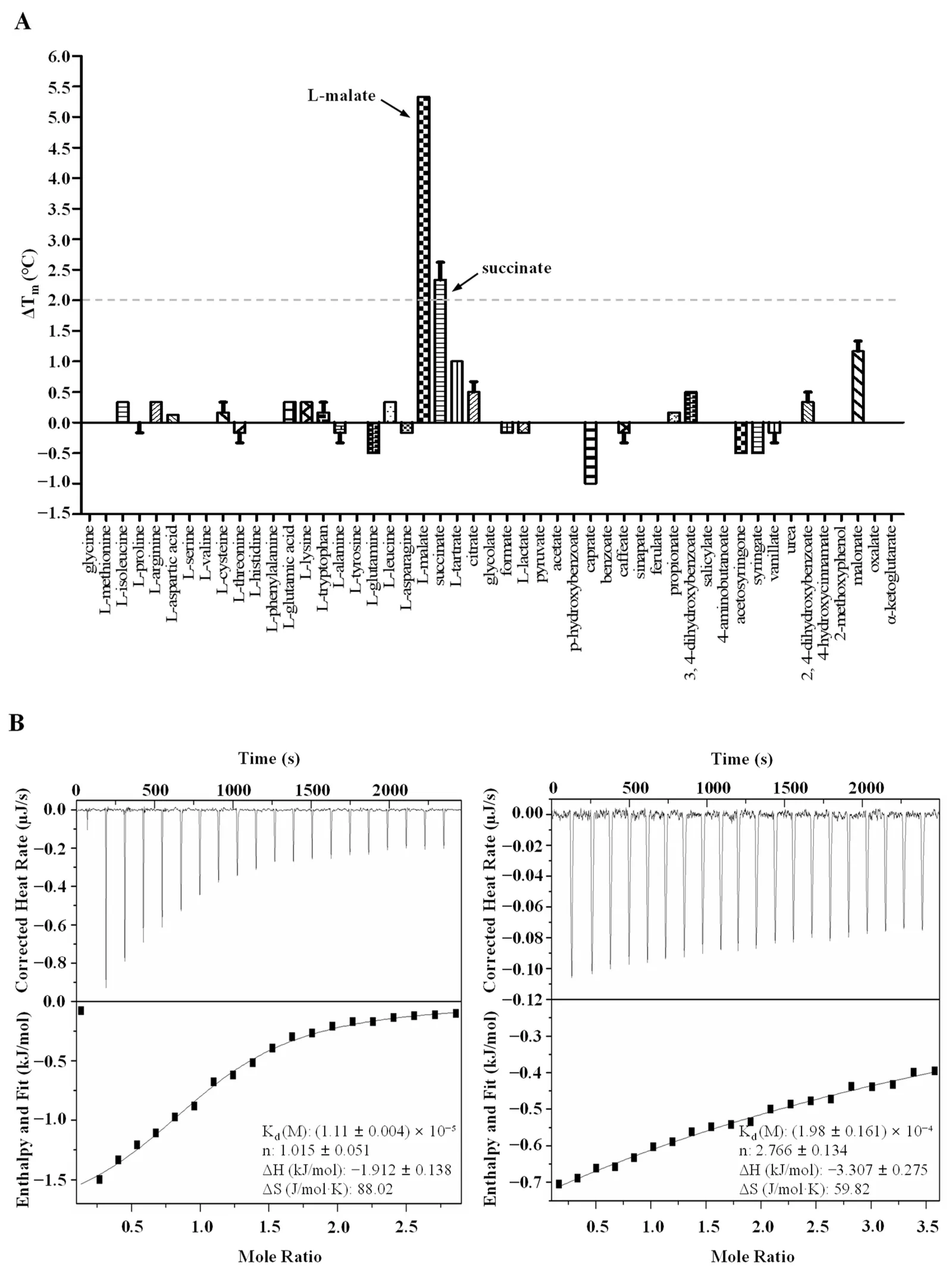
Identification of the ligands of LBD_0646_. (**A**) ΔT_m_ values of LBD_0646_ upon addition of each tested compound. T_m_ values of LBD_0646_ were determined by monitoring the thermal denaturation curve using differential scanning fluorimetry (DSF). ΔT_m_ values were calculated as the T_m_ of LBD_0646_ in the presence of the tested compound minus that of the purified protein alone. DSF was performed in triplicate and data were presented in the mean ± standard deviation. (**B**) Isothermal titration calorimetry (ITC) of LBD_0646_ with two ligands. The upper panels show the raw titration data and the lower panels show the integrated titration data fitted with the “Independent site” model of ORIGIN. The shown figures are results of representative experiments. The dissociation constant (K_d_), stoichiometry of binding of ligands to LBD_0646_ (n), enthalpy change (ΔH), and entropy change (ΔS) derived from the fitted curves are means and standard deviations from three experiments. The left panels show the results of LBD_0646_ titrated with 1 mmol/L L-malate. The right panels show the results of LBD_0646_ titrated with 1 mmol/L succinate.

Figure 3 also shows that complementation with the Atu0646-expressing plasmid rendered the chemotactic responses of *atu0646*-deletted *A. tumefaciens* strain to both L-malate and succinate significantly stronger than those of wild type strain C58 to both ligands, indicating that the function of Atu0646 can be fully complemented by an Atu0646-expressing plasmid and that this plasmid complementation approach can be used for further elucidation of the ligand-binding mechanism of LBD_0646_.

**Figure 3:**
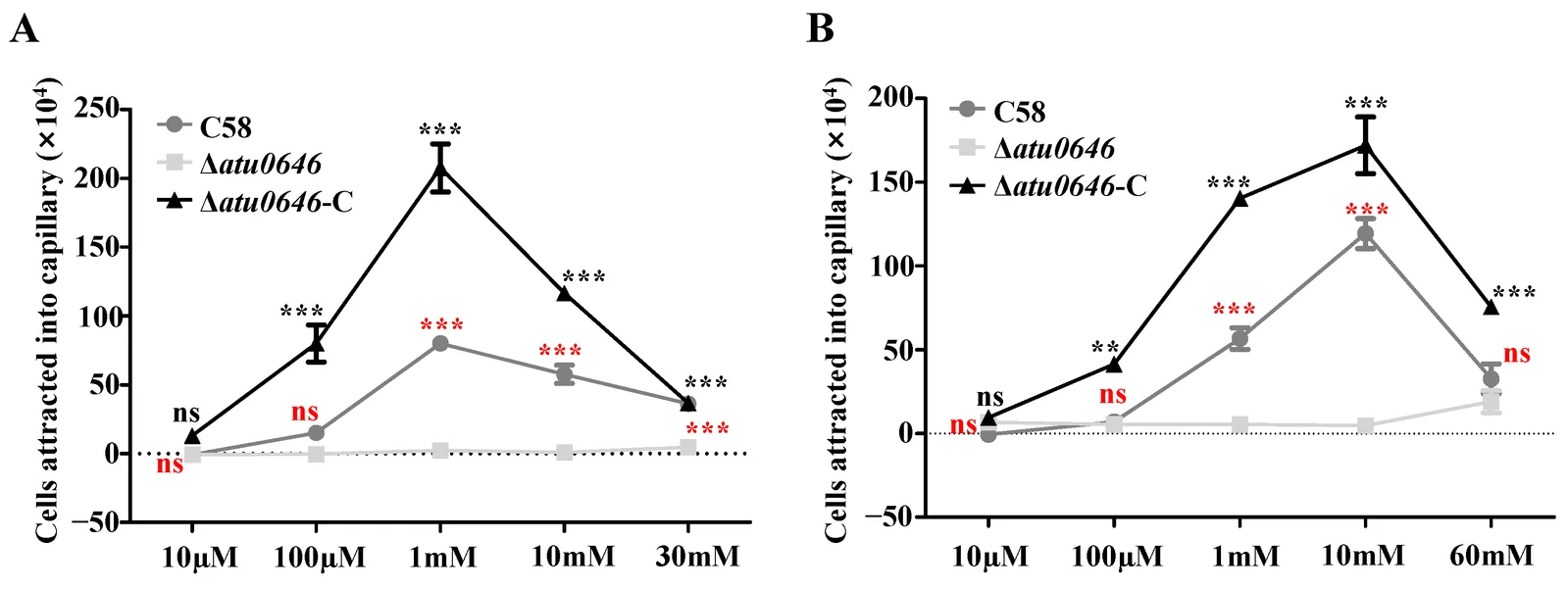
Effect of *atu0646* deletion on the chemotactic responses of *A. tumefaciens* to L-malate and succinate. Chemotactic responses were quantitatively determined using capillary chemotaxis assays. Three different *A. tumefaciens* strains: wild-type (C58), *atu0646*-deletion mutant (Δ*atu0646*), and *atu0646*-deletion mutant complemented with an Atu0646-expressing plasmid (Δ*atu0646*-C), were cultured to mid-log phase. Bacterial cells were collected from these cultures, washed with chemotaxis buffer, and resuspended in the buffer to an OD_600nm_ of 0.1. Capillary tubes containing L-malate (**A**) or succinate (**B**) with the indicated concentrations were inserted into the bacterial cell suspensions to allow bacterial cells to migrate into the capillary tubes via chemotaxis. Capillary tubes with buffer alone served as the control. Migrated bacterial cells inside capillaries were enumerated by colony counting. The numbers of cells attracted into ligand-containing capillaries were normalized by subtracting the average cell numbers recovered from buffer-only control capillaries. Data were presented as the mean ± standard deviation of three independent biological replicates, with three technical replicates conducted per biological sample. Asterisks indicate *p* values (ns, not significant; ** <0.01; *** <0.001) determined by Student’s *t*-test. Statistical comparisons were performed against Δ*atu0646* at identical ligand concentrations to assess significant differences.

### 3.3. Analysis of Key Amino Acid Residues for the Function of Atu0646

Since the ligands of Atu0646 are L-malate and succinate, we first searched for the LBDs that can bind L-malate or succinate and compared their structures. At least 13 distinct LBDs have been reported to bind or sense L-malate or succinate [46,47,48,49,50,51,52,53,54,55,56,57]. However, these 13 LBDs belong to five different structural types, including dCache_1, sCache_2, sCache_3_2, 4HB_MCP_1, and HBM (helical bimodular) [23]. LBD_0646_ was predicted to belong to the sCache_2 type and was thus compared with other sCache_2-type LBDs that can bind or sense L-malate or succinate. Sequence alignment of LBDs from these C4-dicarboxylate chemoreceptors showed that up to 17 residues are identical across these sCache-type LBDs (Supplementary Figure S2), while none of their ligand-binding mechanisms has been resolved [55,56,57]. Therefore, sequence analysis provides very limited information for elucidating the ligand-binding mechanism.

However, crystal structures of three sCache-type LBDs that bind C2 (glycolate and acetate) or C3 (propionate and pyruvate) carboxylates have been resolved and can serve as templates for building the three-dimensional structural model of LBD_0646_ [45,58,59]. The built LBD_0646_ model was used for molecular docking simulations with the ligands L-malate and succinate via AutoDock Vina. Molecular docking predicted that at least three distinct pockets of different sizes on the surface of LBD_0646_ could bind these two ligands with different binding energies. By combining structural comparisons with the templates for building the model and analysis of binding energy values (where more negative values indicate stronger binding affinity), the pocket corresponding to the ligand-binding pockets of the templates (PDB ID 5G4Z, 5G4Y, 4K08, 2QHK, 4EXO) was proposed as the functional ligand-binding pocket of LBD_0646_ for both L-malate (Figure 4A) and succinate (Figure 4B). Even in the pocket with the highest potential for ligand binding, these docking results indicate that the ligands can bind in two distinct modes (I and II in Figure 4). Furthermore, the binding energies of these two modes are highly similar, making it difficult to determine the exact binding mode of the ligand. Therefore, we regard the residues possibly involved in both binding modes as key residues, including Y90, H103, R108, S119, Y124, R125, P144, N151, K155, and Y174 (Figure 4).

Considering that the importance of the above-mentioned residues for ligand binding differs, we chose 8 residues, Y90, H103, R108, S119, R125, N151, K155, and Y174, for site-directed substitution. Based on the properties and bulkiness of amino acid side chains as well as the location of residues in the binding pocket, the 8 residues were substituted with different amino acids: Y90, H103, and K155 with alanine; R108, S119, R125, and N151 with tyrosine; and Y174 with lysine. All eight residues were individually constructed into single-residue-substitution variants, thus generating 8 LBD_0646_ variants: LBD_0646_-Y90A, LBD_0646_-H103A, LBD_0646_-K155A, LBD_0646_-R108Y, LBD_0646_-S119Y, LBD_0646_-R125Y, LBD_0646_-N151Y, and LBD_0646_-Y174K.

The thermal denaturation of these eight purified LBD_0646_ variants was tested using DSF (Supplementary Figure S3). Five variants (LBD_0646_-Y90A, LBD_0646_-H103A, LBD_0646_-K155A, LBD0_646_-R125Y, and LBD_0646_-Y174K) exhibited abnormal denaturation curves and the addition of L-malate and succinate did not affect their denaturation curves. Three variants (LBD_0646_-R108Y, LBD_0646_-S119Y, and LBD_0646_-N151Y) displayed normal thermal denaturation curves. The increases in melting temperature (ΔT_m_) of these three variants upon the addition of L-malate and succinate are shown in Figure 5. The ΔT_m_ values of both LBD_0646_-R108Y and LBD_0646_-N151Y upon the addition of either L-malate or succinate are smaller than those of wild-type LBD_0646_, suggesting that the complexes formed by these two LBD_0646_ variants with their ligands are less stable than those formed by wild-type LBD_0646_. In contrast, the ΔT_m_ values of LBD_0646_-S119Y upon the addition of either L-malate or succinate are substantially larger than those of wild-type LBD_0646_, demonstrating that the complexes of this variant with its ligands are more stable than those of LBD_0646_.

To further testify the roles of these residues in in vivo chemotaxis, 6 corresponding Atu0646 variants (Atu0646-Y90A, Atu0646-H103A, Atu0646-K155A, Atu0646-R108Y, Atu0646-S119Y, and Atu0646-N151Y) and wild-type Atu0646 were individually introduced into the *atu0646*-deleted *A. tumefaciens* strain via plasmid complementation. As shown in Figure 6, three Atu0646 variants (Atu0646-Y90A, Atu0646-H103A, and Atu0646-K155A) completely failed to restore the chemotactic responses of *A. tumefaciens* to both L-malate and succinate. Both Atu0646-R108Y and Atu0646-N151Y could partially restore the chemotactic responses of the *atu0646*-deficient strain to L-malate and succinate, whereas Atu0646-S119Y significantly enhanced the chemotactic response to both ligands compared with wild-type Atu0646 complementation. These in vivo chemotactic results are consistent with the in vitro LBD DSF results.

The consistent results from the in vivo chemotaxis assay and the in vitro LBD DSF assay prompted us to further quantify the binding affinities of LBD_0646_-S119Y for L-malate and succinate. As shown in Figure 7, the K_d_ values for LBD_0646_-S119Y–L-malate (6.44 ± 0.46 μmol/L) and LBD_0646_-S119Y–succinate (26.6 ± 1.95 μmol/L) complexes were markedly lower than those of wild-type LBD_0646_ bound to L-malate (11.1 ± 0.04 μmol/L) and succinate (198 ± 16.1 μmol/L). These data suggested that LBD_0646_-S119Y possesses enhanced binding affinity toward both ligands relative to wild-type LBD_0646_, which aligns with the in vivo chemotaxis data presented in Figure 6. Chemotactic responses mediated by Atu0646-S119Y across gradients of L-malate and succinate further verified that this residue substitution enhanced the chemotactic responses of the Atu0646 chemoreceptor to both ligands over a wide range of ligand concentrations (Figure 8).

The enhanced ligand-binding capability of LBD_0646_ caused by the S119Y substitution also helps us clarify the exact binding mode of the ligand. We constructed a structural model of LBD_0646_-S119Y and performed molecular docking for the two ligand binding modes based on this model (Supplementary Figure S4). The docking results revealed that binding mode II of L-malate was altered, accompanied by a less negative binding energy and binding mode II of succinate was abolished by S119Y substitution. In contrast, binding mode I remained largely unchanged and exhibited a more negative binding energy, which is consistent with the improved binding affinity. Accordingly, it can be predicted with greater confidence that the ligand binds via mode I rather than mode II.

### 3.4. S119Y Substitution Broadens the Ligand Spectrum of Atu0646

Since substitution of S119 with Y altered the binding affinities of Atu0646 for its two cognate ligands, we next tested whether this Atu0646 variant could broaden its ligand-binding range. We then screened the chemicals in Figure 2 for their effects on the thermal denaturation of LBD_0646_-S119Y using DSF, aiming to identify additional ligands of LBD_0646_-S119Y. It was found that L-aspartate affected the thermal denaturation of LBD_0646_-S119Y: a 1 °C increase in T_m_ was observed for LBD_0646_-S119Y upon L-aspartate supplementation, whereas no obvious T_m_ shift was detected for wild-type LBD_0646_ under the same treatment (Supplementary Figure S5). This observation suggested that the S119Y substitution enables LBD_0646_ to recognize L-aspartate. We further performed ITC to quantify the binding affinities of wild-type LBD_0646_ and LBD_0646_-S119Y for L-aspartate. As presented in Figure 9A, LBD_0646_ exhibited no binding to L-aspartate. In contrast, LBD_0646_-S119Y formed a stable complex with L-aspartate, with a dissociation constant (K_d_) of 1.06 ± 0.12 μmol/L. Encouraged by these in vitro binding data, we then explored whether Atu0646-S119Y could trigger L-aspartate-dependent chemotaxis in *A. tumefaciens* using quantitative capillary chemotaxis assays. Only the strain complemented with Atu0646-S119Y displayed robust chemotaxis toward L-aspartate; neither wild-type C58 nor the strain complemented with wild-type Atu0646 (Δ*atu0646*-C) exhibited an obvious chemotactic response to L-aspartate. These data supported that the S119Y substitution enables Atu0646 to recognize L-aspartate. Relative to the Δ*atu0646* strain (negative control), C58 and Δ*atu0646*-C appeared to show a slight negative response at some tested L-aspartate concentrations (Figure 9B). However, this does not undermine our conclusion that the S119Y substitution expands the ligand spectrum of Atu0646.

## 4. Discussion

The key to the functional characterization of chemoreceptors lies in the identification of their cognate ligands, since different chemoreceptors share highly similar mechanisms in signal transmission and the regulation of downstream protein functions. For transmembrane chemoreceptors, a ligand binds to the periplasmic LBD and induces a conformation change. Subsequently this ligand-induced conformational change signal is transmitted to the CSD through the transmembrane helices [13,14]. Most periplasmic LBDs exist as highly autonomous domains in structure and can be expressed as a standalone protein and maintain a stable conformation in solution although a small number of periplasmic LBDs fail to do so [34]. Atu0646 chemoreceptor carries a typical sCache-type LBD. Like most periplasmic LBDs, the recombinant LBD of Atu0646 adopts a stable conformation in solution and can be used to screen its cognate ligands. Results from both DSF and ITC showed that LBD_0646_ could directly bind both L-malate and succinate. The *atu0646* deletion experiment confirmed that Atu0646 functions as a receptor for both L-malate and succinate in *A. tumefaciens* C58. From the in vitro screening of potential ligands for the LBD to the in vivo determination of chemotactic responses following deletion of the entire chemoreceptor, comprehensive and highly convincing experimental evidence was provided for the functional characterization of Atu0646. Accordingly, following the common workflow for functional identification of chemoreceptors, we identified an additional chemoreceptor and determined its ligands. This again demonstrates that the strategy from in vitro screening of potential LBD ligands to the characterization of chemotactic responses in chemoreceptor deletion mutants is an effective approach for the functional characterization of most chemoreceptors.

Functional characterization of the Atu0646 chemoreceptor expands our knowledge of *A. tumefaciens*’s sensory repertoire. The two ligands sensed by Atu0646 are tricarboxylic acid (TCA) cycle intermediates, L-malate and succinate, which are abundant in plant tissues, root exudates [49,56], and bleeding sap [60]. Most plant-derived carboxylates act as carbon and energy sources for *A. tumefaciens* via the TCA cycle [61]. As a facultative pathogen, *A. tumefaciens* predominantly adopts a saprophytic lifestyle and encounters a broad spectrum of environments, including bulk soil and the plant rhizosphere. As a phytopathogen, it infects most dicotyledonous plants via host wound sites [26]. Atu0646-mediated chemotactic attraction toward L-malate and succinate benefits *A. tumefaciens* during rhizosphere colonization and the localization of susceptible wound sites, given that both root exudates and wound bleeding sap contain high levels of L-malate and succinate [56,60].

A detailed elucidation of the chemoreceptor-ligand binding mechanism requires the high-resolution three-dimensional structure of the chemoreceptor, or at minimum the detailed three-dimensional structure of its LBD. In this study, in the absence of three-dimensional structural information for the LBD of the Atu0646 chemoreceptor, we built a three-dimensional structural model of LBD_0646_ using AlphaFold for homology modeling. Molecular docking was then performed to predict the ligand-binding pocket of LBD_0646_, based on which key amino acid residues potentially involved in ligand binding were identified. Site-directed mutagenesis was subsequently carried out to verify the roles of these residues in the ligand-binding function of the chemoreceptor. However, although site-directed mutagenesis of amino acid residues can establish the importance of individual residues in chemoreceptor-ligand binding, it cannot distinguish whether their effects on ligand-binding affinity arise indirectly from conformational changes or directly from altered interactions with the ligand. DSF might be helpful for us to determine whether a residue-substitution causes protein unfolding. Five residue-substitutions (Y90A, H103A, K155A, R125Y, and Y174K) caused LBD_0646_ to display abnormal thermal denaturation curves, and three corresponding residue-substitution Atu0646 chemoreceptor variants (Y90A, H103A, and K155A) were further verified to have completely lost their function in in vivo chemotaxis assays. Such results evidently provide limited additional information for the in-depth analysis of the ligand-binding mechanism. The LBD_0646_ variants with the other three residue substitutions (R108Y, S119Y, and N151Y) exhibited normal thermal denaturation curves, indicating that they can form stable conformations. The corresponding Atu0646 variants displayed impaired function to different extents in in vivo chemotaxis assays. These results combined with their ITC data can solidly confirm that these three residues are directly involved in the ligand binding, verifying the reliability of the built model and molecular docking.

The construction of the LBD model and the elucidation of its ligand-binding mechanism lay the foundation for the engineering of chemoreceptors, enabling us to predict how substitutions of residues involved in ligand binding may affect ligand recognition and binding. In fact, it is relatively difficult to predict the exact binding sites on the chemoreceptor surface for small flexible molecules such as L-malate, which contain multiple charged or polar groups. This is because the chemoreceptor surface also possesses numerous charged or polar amino acid side chains, which can easily form intermolecular interactions with such multifunctional small molecules. As a result, these ligands tend to interact non-specifically at multiple positions rather than a single defined site. Therefore, when performing molecular docking of L-malate and succinate with LBD_0646_, multiple potential binding sites on its surface were predicted. Even within the binding pocket shown in Figure 2, each ligand displays two distinct possible binding modes. Residue S119 is located at the edge of the ligand-binding pocket. When replaced with tyrosine, which also contains a hydroxyl group but has a larger side chain, the binding pocket may become more compact and fit the ligand better, making the ligand less likely to dissociate after binding. Thus, we observed that S119Y substitution improved the binding affinity of Atu0646 for L-malate and succinate. L-aspartate shares the same C4-dicarboxylate structural backbone as L-malate. Although their respective side-chain groups differ (one is -OH and the other is -NH_2_), both groups readily form hydrogen bonds with other polar groups under appropriate pH conditions. Therefore, the S119Y substitution has the potential to engineer Atu0646 into a chemoreceptor capable of recognizing L-aspartate. The experimental results confirmed our prediction and successfully achieved the protein engineering of Atu0646.

## 5. Conclusions

*Agrobacterium tumefaciens atu0646*-encoded protein Atu0646 is a sCache-type chemoreceptor and serves as the only chemoreceptor for L-malate and succinate in *A. tumefaciens* C58. The truncated periplasmic LBD of Atu0646 can maintain its native conformation and is suitable for screening its potential ligands. The LBD_0646_ model built by AlphaFold, together with molecular docking results, provides reliable information for elucidating the ligand-binding mechanism of LBD_0646_. Based on the molecular docking predictions, 8 residues (Y90, H103, R108, S119, R125, N151, K155, and Y174) were selected for site-directed mutagenesis. Five residue-substituted LBD_0646_ variants (Y90A, H103A, K155A, R125Y, and Y174K) completely lost their function due to the alteration of the LBD structure. Two residue-substituted LBD variants (R108Y and N151Y) did not disrupt the LBD structure but impaired its ligand-binding affinity. A more important finding is that the S119Y mutation not only enhanced binding to both cognate ligands but also conferred novel ligand specificity toward L-aspartate. The successful expansion of the functional scope of the Atu0646 chemoreceptor demonstrates that a single-point mutation can reshape the ligand specificity of chemoreceptors. This finding not only provides valuable insights into chemoreceptor-ligand recognition mechanisms and advances our understanding of chemoreceptor structure-function relationship, but also offers promising applications for the design and engineering of novel biosensors.

## Figures and Tables

**Figure 1:**
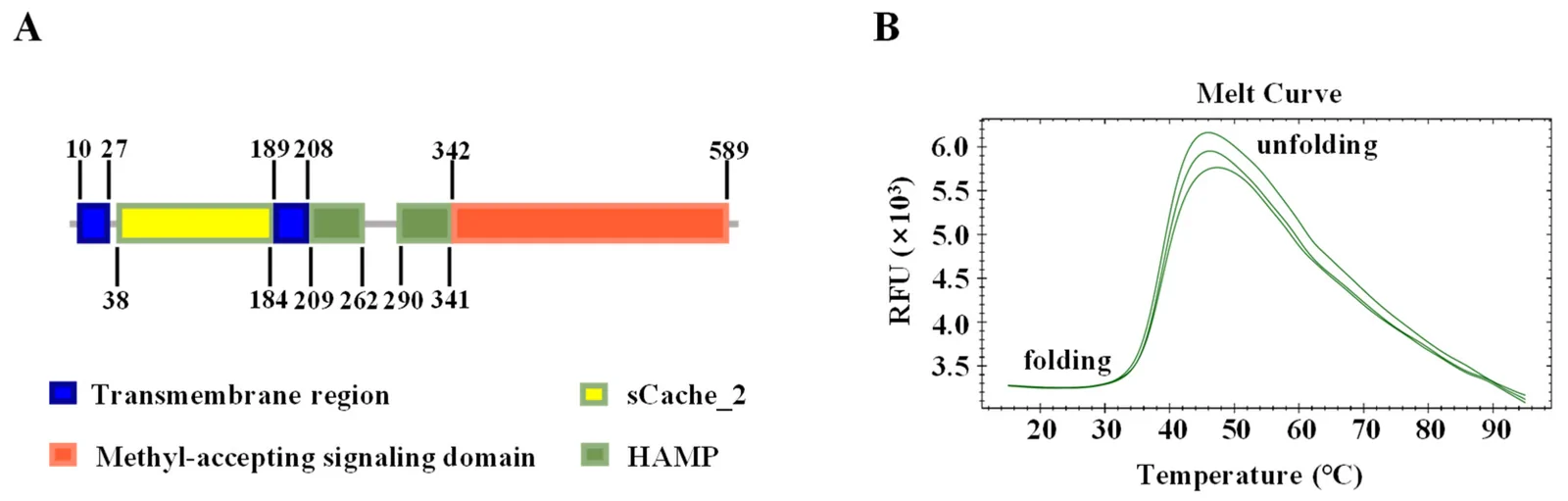
Domain architecture analysis of Atu0646 and thermal denaturation curve of the purified LBD_0646_. (**A**) Domain architecture analysis of Atu0646. Domain architecture analysis was performed using the KEGG (https://www.kegg.jp/kegg/kegg2.html; accessed on 13 March 2026) and SMART (https://smart.embl.de/; accessed on 13 March 2026) databases. Different domains of Atu0646 were represented by distinct colored shapes, with numerical annotations indicating the start and end residue positions of each domain. (**B**) Thermal denaturation curve of the purified LBD_0646_. Residues 27–189 were expressed as a 6×His tagged recombinant protein (LBD_0646_). The thermal denaturation of the purified LBD_0646_ was monitored by differential scanning fluorimetry (DSF). DSF was performed in triplicate.

**Figure 4:**
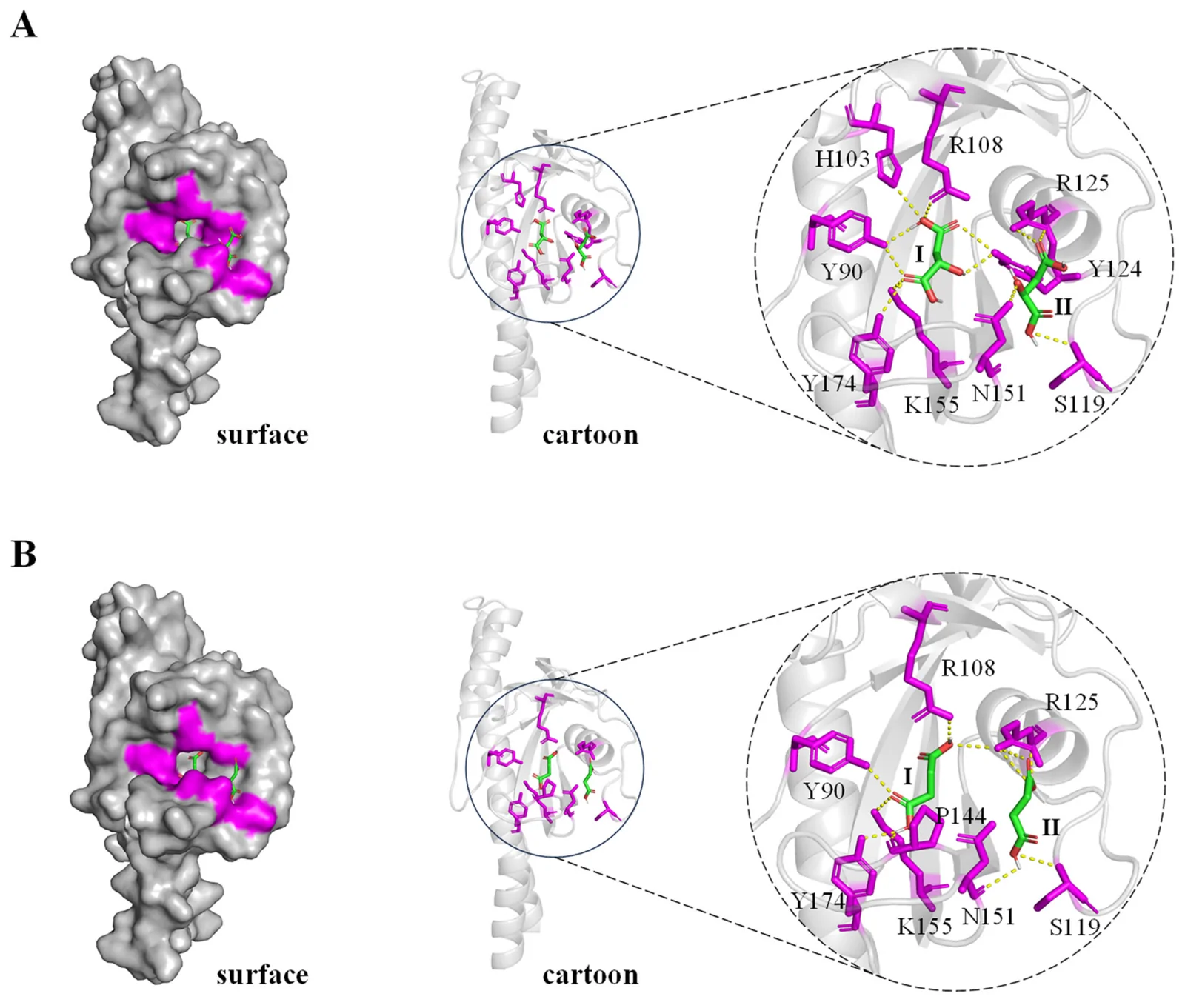
Ligand-binding models of LBD_0646_. The structural model of LBD_0646_ was built by AlphaFold and the docking of L-malate (**A**) and succinate (**B**) into LBD_0646_ was performed using AutoDock Vina. Modeling results are presented in surface and cartoon representations. The ligand-binding sites are zoomed in in the rightmost panels to show two binding modes (I and II). Ligands are shown as green sticks. Magenta regions (stick representation) indicate key residues involved in ligand binding, and yellow dashed lines represent the predicted interactions between the ligand and surrounding residues. Residue labels are shown using the standard one-letter amino acid code and residue numbering.

**Figure 5:**
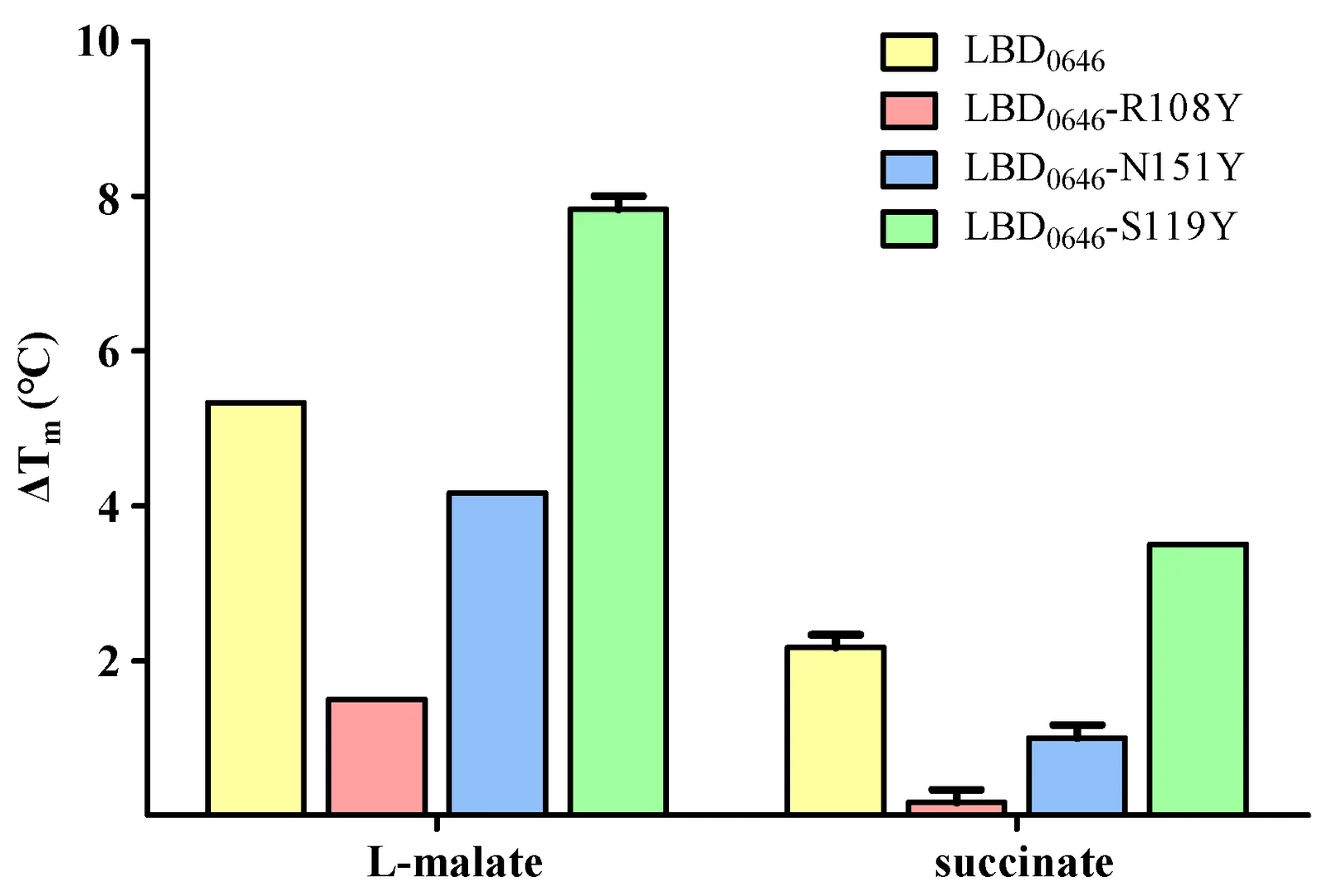
Effects of key residue substitutions on the structural stability and ligand-binding capability of LBD_0646_. Eight LBD_0646_ variants (LBD_0646_-Y90A, LBD_0646_-H103A, LBD_0646_-K155A, LBD_0646_-R108Y, LBD_0646_-S119Y, LBD_0646_-R125Y, LBD_0646_-N151Y, and LBD_0646_-Y174K) were expressed as N-terminal 6×His-tagged recombinant proteins and purified using a Ni-resin column. Thermal denaturation of the purified LBD_0646_ variants was monitored by DSF, and the resulting curves were used to assess their structural stability. The thermal denaturation curves of five variants (LBD_0646_-Y90A, LBD_0646_-H103A, LBD_0646_-K155A, LBD_0646_-R125Y, and LBD_0646_-Y174K) were abnormal, and the addition of L-malate and succinate did not affect their denaturation curves. The other three variants possessed normal thermal denaturation profiles and their ligand-binding capabilities were further evaluated by measuring the T_m_ increments (ΔT_m_) induced by ligand addition. Thermal denaturation curves of all eight variants are presented in Figure S3. Only the ΔT_m_ values of three variants (LBD_0646_-R108Y, LBD_0646_-S119Y, and LBD_0646_-N151Y) upon addition of 1 mmol/L L-malate or 1 mmol/L succinate are presented here. All DSF assays were performed in triplicate, and data are presented as mean ± standard deviation.

**Figure 6:**
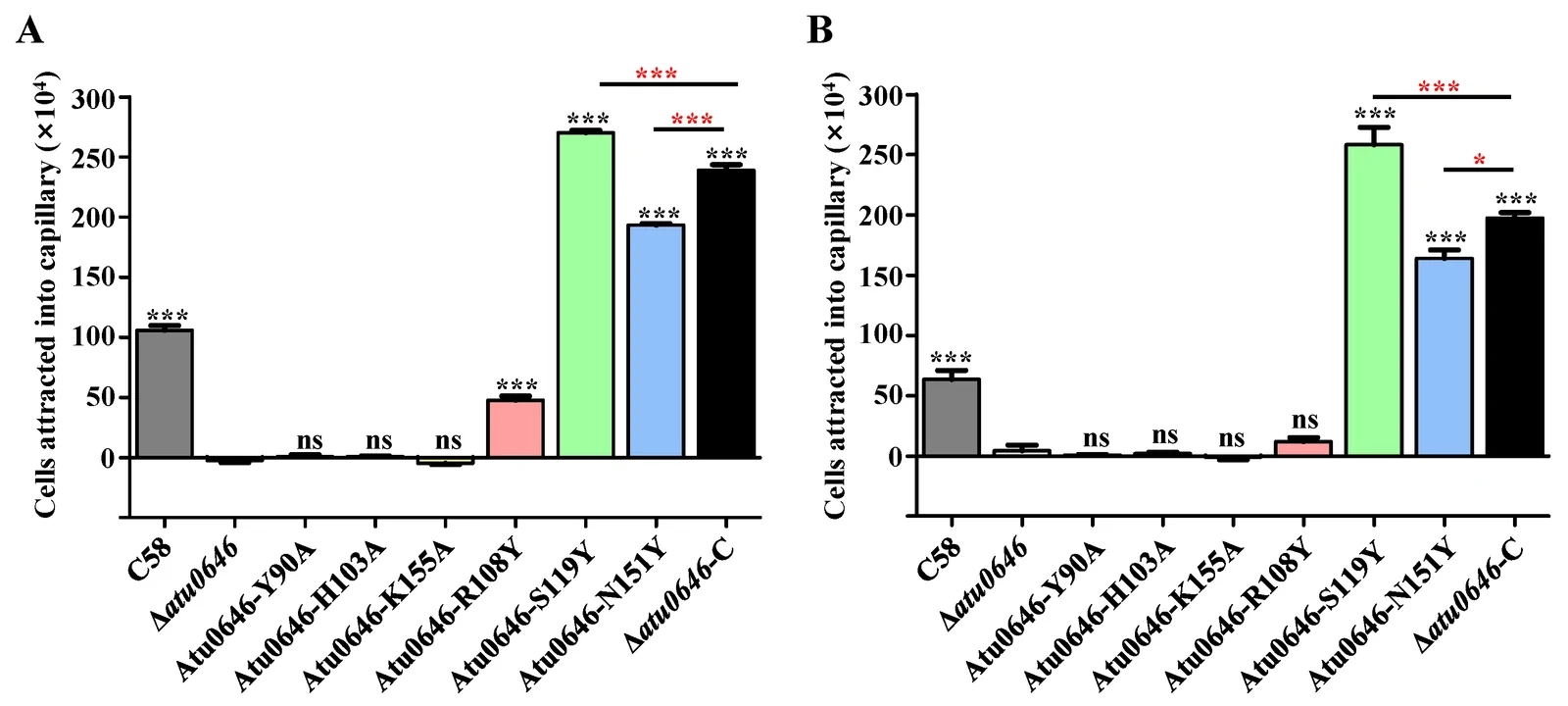
Effects of key residue substitutions on the function of Atu0646. Six Atu0646 variants (Atu0646-Y90A, Atu0646-H103A, Atu0646-K155A, Atu0646-R108Y, Atu0646-S119Y, and Atu0646-N151Y) were used to complement the Atu0646-deficient mutant (Δ*atu0646*) via the introduction of Atu0646 variant-expressing plasmids. The capillary chemotaxis assay was used to quantitatively test the chemotactic responses of the complemented strains to 1 mmol/L L-malate (**A**) or 1 mmol/L succinate (**B**). Capillary tubes with buffer alone served as the control. The numbers of cells attracted into ligand-containing capillaries were normalized by subtracting the average cell numbers recovered from buffer-only control capillaries. Data were presented as the mean ± standard deviation of three independent biological replicates, with three technical replicates performed for each biological sample. Asterisks denote *p* values (ns, not significant; * *p* < 0.05; *** *p* < 0.001) calculated via Student’s *t*-test. In addition to statistical comparisons between every strain and the Δ*atu0646* mutant to identify significant differences (marked by black ns or asterisks), pairwise statistical comparisons were also performed for strains connected by lines (marked by red ns or asterisks).

**Figure 7:**
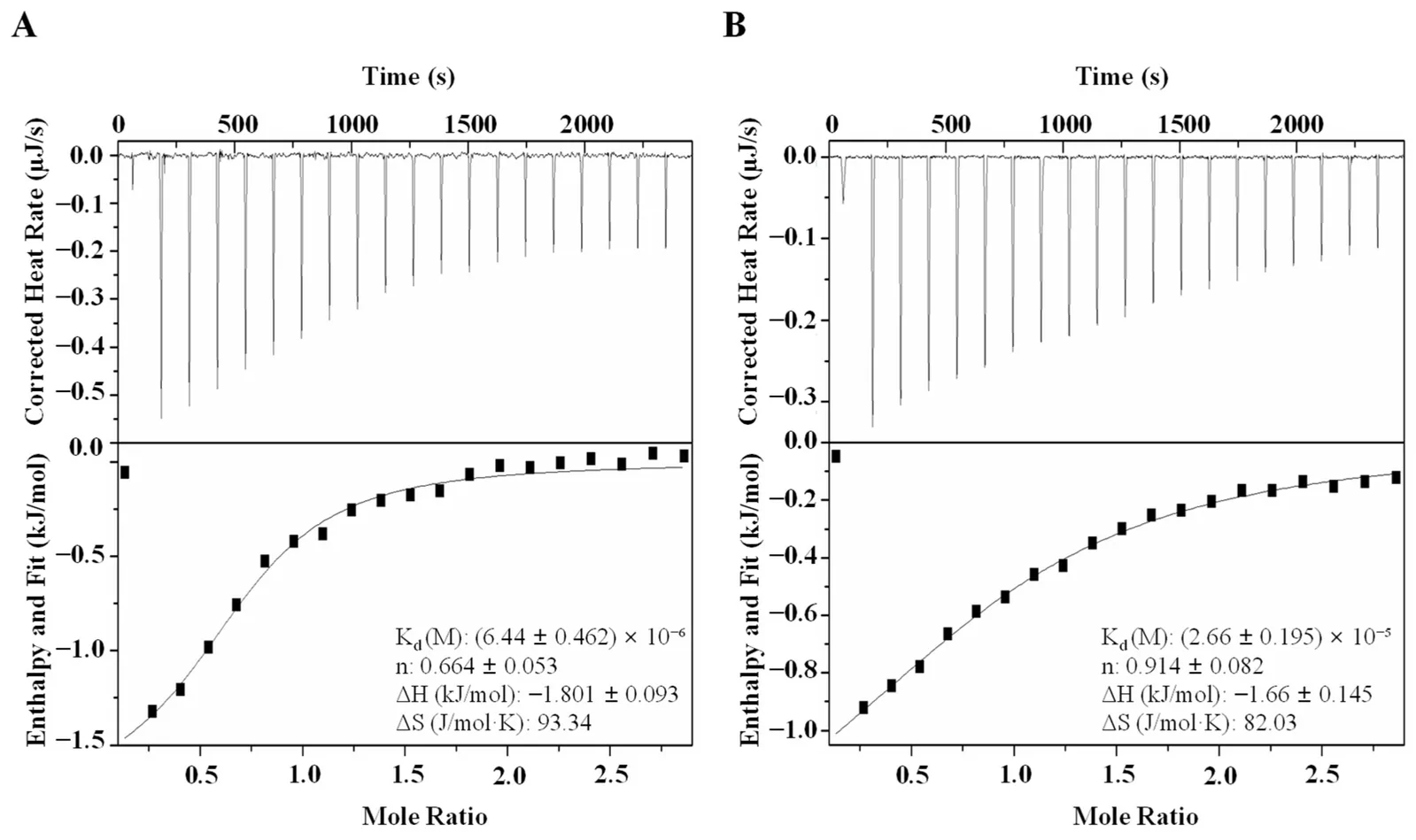
Affinities of LBD_0646_ variant (LBD_0646_-S119Y) for L-malic acid and succinate. The purified LBD_0646_-S119Y protein was titrated with 1 mmol/L L-malate (**A**) or 1 mmol/L succinate (**B**). The heat changes in protein-ligand reactions were measured by isothermal titration calorimetry (ITC). The upper panels show the raw titration data and the lower panels show the integrated titration data fitted with the “Independent site” model of ORIGIN. The shown figures are results of representative experiments. The dissociation constant (Kd), stoichiometry of binding of ligands to LBD_0646_-S119Y (n), enthalpy change (ΔH), and entropy change (ΔS) derived from the fitted curves are means and standard deviations from three experiments.

**Figure 8:**
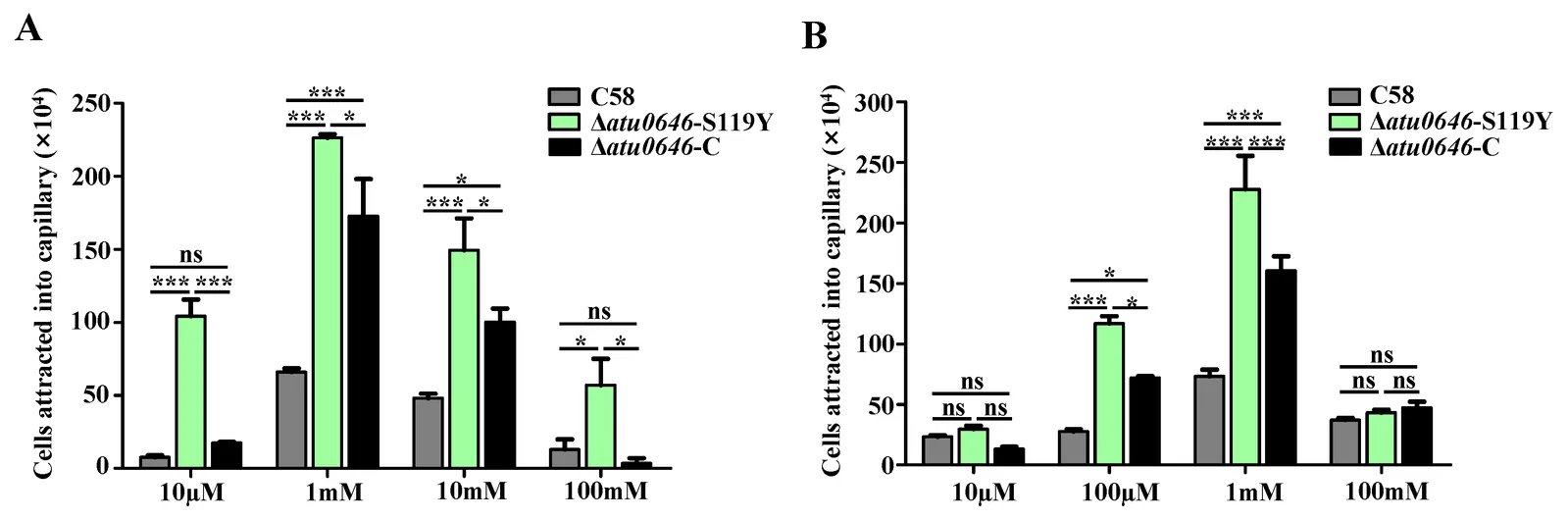
Chemotactic responses of the Atu0646 variant (Atu0646-S119Y) to different ligand concentrations. Bacterial chemotaxis was quantitatively tested using the capillary chemotaxis assay. To compare the Atu0646-S119Y variant with wild-type Atu0646, four *A. tumefaciens* strains (C58, Δ*atu0646*, Δ*atu0646*-C, and Δ*atu0646*-S119Y) were used to test their chemotactic responses to different concentrations of L-malate (**A**) or succinate (**B**). The Δ*atu0646* strain was used as the negative control. The numbers of cells attracted into ligand-containing capillaries were normalized by subtracting the average cell numbers of the negative control strain that migrated into capillaries with identical ligand concentration. Data were presented as the mean ± standard deviation of three independent biological replicates, with three technical replicates conducted per biological sample. Pairwise statistical comparisons between strains were performed at identical ligand concentrations via Student’s *t*-test. Significant differences between compared strains were indicated by the asterisks above the horizontal lines connecting them. Asterisks represent significance thresholds: *p* < 0.05 (*), <0.001 (***), not significant (ns). C58: wild-type strain; Δ*atu0646*: *atu0646*-deficient mutant; Δ*atu0646*-C: Δ*atu0646* complemented with wild-type Atu0646; Δ*atu0646*-S119Y: Δ*atu0646* complemented with Atu0646 variant (Atu0646-S119Y).

**Figure 9:**
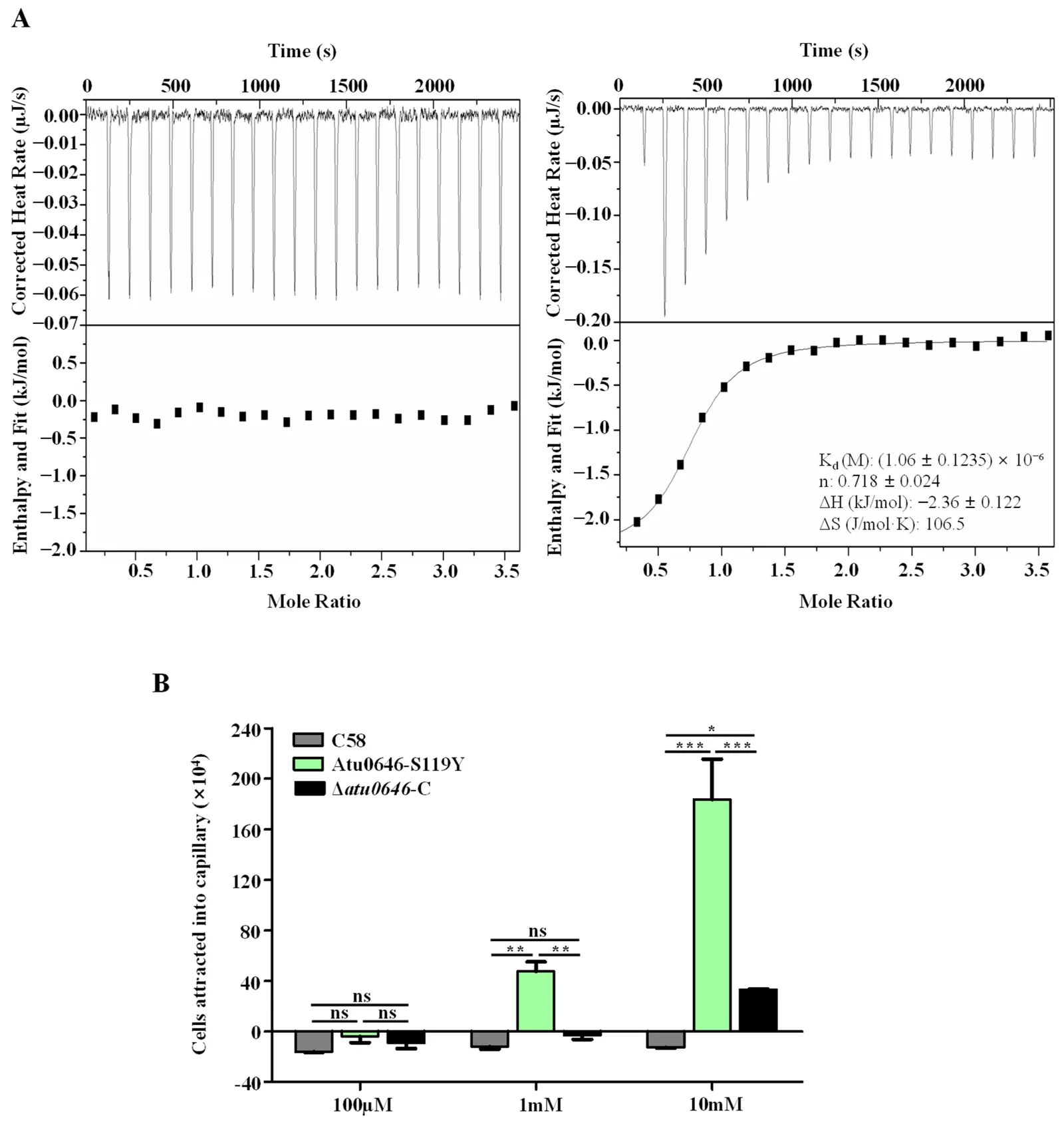
S119Y substitution makes Atu0646 bind and sense L-aspartate. The binding capability of LBD_0646_-S119Y to Asp was determined using ITC and the sensing of Asp by Atu0646-S119Y was determined using the capillary chemotaxis assay. (**A**) ITC assay of binding affinities for Asp. LBD_0646_ (left panel) and LBD_0646_-S119Y (right panel) were titrated with 1mmol/L Asp by ITC. The upper panels show the raw titration data and the lower panels show the integrated titration data fitted with the “Independent site” model of ORIGIN. The shown figures are results of representative experiments. The dissociation constant (Kd), stoichiometry of binding of Asp to LBD_0646_-S119Y (n), enthalpy change (ΔH), and entropy change (ΔS) derived from the fitted curves are means and standard deviations from three experiments. (**B**) Chemotactic responses to Asp. Four *A. tumefaciens* strains (C58, Δ*atu0646*, Δ*atu0646*-C, and *Δatu0646*-S119Y) were used to test their chemotactic responses to different concentrations of Asp. Bacterial chemotaxis was quantitatively determined using the capillary chemotaxis assay. The Δ*atu0646* strain was used as the negative control. The numbers of cells attracted into Asp-containing capillaries were normalized by subtracting the average cell numbers of the negative control strain that migrated into capillaries with identical concentration of Asp. Data were presented as the mean ± standard deviation of three independent biological replicates, with three technical replicates conducted per biological sample. Statistical comparisons between strains were performed at identical Asp concentrations via Student’s *t*-test. Asterisks labelled above the line connecting the compered strains represent significance thresholds: *p* < 0.05 (*), <0.01 (**), <0.001 (***), not significant (ns). Strain names are the same as in Figure 8.

## Data Availability

The original contributions presented in this study are included in the article and supplementary material. Further inquiries can be directed to the corresponding author.

## Supplementary Materials

The following supporting information can be downloaded at https://www.mdpi.com/article/10.3390/biom16101405/s1, Table S1: Bacterial strains and plasmids used in this study [38,62,63,64,65]; Table S2: Primer sequences used in this study; Figure S1: Concentration gradient binding validation for L-malate and succinate; Figure S2: Sequence alignment of 4 sCache_2-type LBDs from C4-dicarboxylic acid chemoreceptors; Figure S3: Thermal denaturation curves of 8 LBD_0646_ variants; Figure S4: Ligand-binding models of LBD_0646_-S119Y; Figure S5: DSF determination of the binding of L-aspartate to the LBD_0646_ and LBD_0646_-S119Y variant.

## Abbreviations

The following abbreviations are used in this manuscript:

MCP	Methyl-accepting chemotaxis protein
LBD	Ligand-binding domain
DSF	Differential scanning fluorimetry
ITC	Isothermal titration calorimetry
CSD	Cytoplasmic signaling domain
TM	Transmembrane helix
MHB	Methylation helix bundle
