# Supplementary material for: Identification and Ligand-Binding Mechanism Analysis of a C4-Dicarboxylate Chemoreceptor from Agrobacterium tumefaciens
Source: Biomolecules. 2026 Sep 27;16(10):1405. doi: 10.3390/biom16101405 (PMC13702588; doi:10.3390/biom16101405)
Supplement: Supplementary file 1 [file biomolecules-16-01405-s001.zip › biomolecules-4518503-supplementary.pdf]

Table S1. Bacterial strains and plasmids used in this study.

Strains or Plasmids	Description	Source
**Strains**		
Escherichia coli		
DH5α	*EndA1 hsdR17 supE44 thi-1 recA1 gyrA96 relA1 (argF-lacZYA)* U169 φ80d*lacZ*, for DNA cloning	Bethesda Research Laboratories
BL21(DE3)	*F^- ompT hsdSB(rB^- mB^-) gal dcm (DE3),* for protein expression	[62]
LBD_{0646}	BL21(DE3) strain with pET30a-LBD_{0646} was transformed in	This study
LBD_{0646}-Y90A	BL21(DE3) with pET30a-Y90A transformed in	This study
LBD_{0646}-H103A	BL21(DE3) with pET30a-H103A transformed in	This study
LBD_{0646}-R108Y	BL21(DE3) with pET30a-R108Y transformed in	This study
LBD_{0646}-S119Y	BL21(DE3) with pET30a-S119Y transformed in	This study
LBD_{0646}-R125Y	BL21(DE3) with pET30a-R125Y transformed in	This study
LBD_{0646}-N151Y	BL21(DE3) with pET30a-N151Y transformed in	This study
LBD_{0646}-K155A	BL21(DE3) with pET30a-K155A transformed in	This study
LBD_{0646}-Y174K	BL21(DE3) with pET30a-Y174K transformed in	This study
Agrobacterium tumefaciens		
C58	Nopaline type strain; pTiC58, pAtC58	[63]
Δ*CheA*	Derivative of C58 in which *cheA* (*atu0517*) ORF was deleted	This study
Δ*atu0646*	Derivative of C58 in which *atu0646* open reading frame (ORF) was deleted	This study
Δ*atu0646*-C	Δ*atu0646* in which *atu0646* expression was restored by plasmid pCB301-*atu0646*	This study
Atu0646-Y90A	Δ*atu0646* in which *atu0646* expression was restored by plasmid pCB301-Y90A	This study
Atu0646-H103A	Δ*atu0646* in which *atu0646* expression was restored by plasmid pCB301-H103A	This study
Atu0646-R108Y	Δ*atu0646* in which *atu0646* expression was restored by plasmid pCB301-R108Y	This study

Atu0646-S119Y	Δ*atu0646* in which *atu0646* expression was restored by plasmid pCB301-S119Y	This study
Atu0646-R125Y	Δ*atu0646* in which *atu0646* expression was restored by plasmid pCB301-R125Y	This study
Atu0646-N151Y	Δ*atu0646* in which *atu0646* expression was restored by plasmid pCB301-N151Y	This study
Atu0646-K155A	Δ*atu0646* in which *atu0646* expression was restored by plasmid pCB301-K155A	This study
Atu0646-Y174K	Δ*atu0646* in which *atu0646* expression was restored by plasmid pCB301-Y174K	This study
**Plasmids**		
pEX18Km	Derivative of pEX18Tc in which Tc^r was replaced by *nptIII* from pCB301, Km^r, Sur^s	[38, 64]
pEX18Km-*atu0646*	pEX18Km carrying a 1,104 bp fragment, which consisted of 324 bp upstream of *atu0646* and 780 bp downstream of *atu0646*; Km^r	This study
pCB301	A minim binary vector plasmid, Km^r	[65]
pCB301-*atu0646*	pCB301 carrying a 2319 bp *atu0646* expression cassette, which consisted of 495 bp upstream, 1824 bp ORF; Km^r	This study
pCB301-Y90A	pCB301 carrying a 2319 bp *atu0646* expression cassette with single residue-substitution Y90A; Km^r	This study
pCB301-H103A	pCB301 carrying a 2319 bp *atu0646* expression cassette with single residue-substitution H103A; Km^r	This study
pCB301-R108Y	pCB301 carrying a 2319 bp *atu0646* expression cassette with single residue-substitution R108Y; Km^r	This study
pCB301-S119Y	pCB301 carrying a 2319 bp *atu0646* expression cassette with single residue-substitution S119Y; Km^r	This study
pCB301-R125Y	pCB301 carrying a 2319 bp *atu0646* expression cassette with single residue-substitution R125Y; Km^r	This study
pCB301-N151Y	pCB301 carrying a 2319 bp *atu0646* expression cassette with single residue-substitution N151Y; Km^r	This study

pCB301-K155A	pCB301 carrying a 2319 bp *atu0646* expression cassette with single residue-substitution K155A; Km^r	This study
pCB301-Y174K	pCB301 carrying a 2319 bp *atu0646* expression cassette with single residue-substitution Y174K; Km^r	This study
pET30a	Expression vector, Km^r	EMD Biosciences
pET30a-LBD_{0646}	pET30a, carrying 489 bp *atu0646-LBD* ORF; Km^r	This study
pET30a-Y90A	pET30a carrying 489 bp *atu0646-LBD* expression cassette with single residue-substitution Y90A; Km^r	This study
pET30a-H103A	pET30a carrying 489 bp *atu0646-LBD* expression cassette with single residue-substitution H103A; Km^r	This study
pET30a-R108Y	pET30a carrying 489 bp *atu0646-LBD* expression cassette with single residue-substitution R108Y; Km^r	This study
pET30a-S119Y	pET30a carrying 489 bp *atu0646-LBD* expression cassette with single residue-substitution S119Y; Km^r	This study
pET30a-R125Y	pET30a carrying 489 bp *atu0646-LBD* expression cassette with single residue-substitution R125Y; Km^r	This study
pET30a-N151Y	pET30a carrying 489 bp *atu0646-LBD* expression cassette with single residue-substitution N151Y; Km^r	This study
pET30a-K155A	pET30a carrying 489 bp *atu0646-LBD* expression cassette with single residue-substitution K155A; Km^r	This study
pET30a-Y174K	pET30a carrying 489 bp *atu0646-LBD* expression cassette with single residue-substitution Y174K; Km^r	This study

Tc^r, tetracycline resistance; Km^r, kanamycin resistance; Sur^s, sucrose sensitivity.

Table S2 Primer sequences used in this study

**Primers**	**Sequence**	**Description**
Δ*atu0646*-1	5'-GACTCTAGA<u>GGATCC</u>GGGGTTTGGGGGTAAACGG-3'	To amplify the upstream sequence of *atu0646*
Δ*atu0646*-2	5'-CCAAACCTTGCGAGCGCCCTACGCTCCGAGGCTATCAACAAC-3'	

Δ*atu0646*-3	5’-GTTGTTGATAGCCTCGGAGCGTAGGGCGCTCGCAAGGTTTGG-3’	To amplify the downstream sequence of *atu0646*
Δ*atu0646*-4	5’-TGCTGCCAACTCGAGTCCGCCAGCGGCGCTTTT-3’	
pCB301-0646-F	5’-AGAACTAGTGGATCCGCGGCACCCGAGGCACATC-3’	To amplify the sequence of *atu0646* and its promoter
pCB301-0646-R	5’-CTTGATATCGAATTCTCACCCCGCCATGCGCAT-3’	
LBD_{0646}-F	5’-ATCGGATCCGAATTCTACCAGCTGAAATCCGCCAC-3’	To amplify the sequence encoding LBD_{0646}
LBD_{0646}-R	5’-GTGGTGGTGCTCGAGTTACGCTTTCATCACCTCACCACG-3’	
Y90A-F	5’-CTCCCGATGGCGCTCTTTTCGGTTACGACTACGACGT-3’	To amplify the sequence encoding Atu0646 with single residue-substitution Y90A
Y90A-R	5’-CGTAACCGAAAAGAGCGCCATCGGGAGTGAAGCG-3’	
H103A-F	5’-GTGACGATGAAATTCGCTCCCGATACAAAACGGGTCG-3’	To amplify the sequence encoding Atu0646 with single residue-substitution H103A
H103A-R	5’-GTTTTGTATCGGGAGCGAATTTCATCGTCACGTCGTAGTC-3’	
R108Y-F	5’-ATCCCGATACAAAATACGTCGGGGAAAACTACAAGGGTAAG-3’	To amplify the sequence encoding Atu0646 with single residue-substitution R108Y
R108Y-R	5’-GTTTTCCCCGACGTATTTTGTATCGGGATGGAATTTCAT-3’	
S119Y-F	5’-GGTAAGCCCGACTACAAGGGCTTTGCCTATCGTGAC-3’	To amplify the sequence encoding Atu0646 with single residue-substitution S119Y
S119Y-R	5’-GGCAAAGCCCTTGTAGTCGGGCTTACCCTTGTAGTTTT-3’	
R125Y-F	5’-GGCTTTGCCTATTATGACGAACTGGTGCGGCTTG-3’	To amplify the sequence encoding Atu0646 with single residue-substitution R125Y
R125Y-R	5’-GCACCAGTTCGTCATAATAGGCAAAGCCCTTGCTGTC-3’	

N151Y-F	5'-GGCATGGAAGGCTATGATTACGCCAAGAGCTCCTACG-3'	To amplify the sequence encoding Atu0646 with single residue-substitution N151Y
N151Y-R	5'-CTCTTGGCGTAATCATAGCCTTCCATGCCAGGCTT-3'	
K155A-F	5'-CAATGATTACGCCGCGAGCTCCTACGCGCTGGC-3'	To amplify the sequence encoding Atu0646 with single residue-substitution K155A
K155A-R	5'-CGCGTAGGAGCTCGCGGCGTAATCATTGCCTTCCAT-3'	
Y174K-F	5'-TGACCGGCGTTAAGATCGACGATCTGAATGCGC-3'	To amplify the sequence encoding Atu0646 with single residue-substitution Y174K
Y174K-F	5'-TTCAGATCGTCGATCTTAACGCCGGTCACGACCAC-3'	

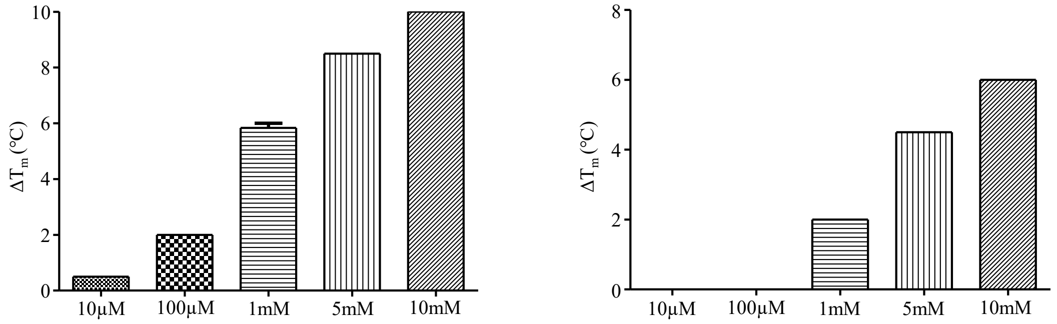

Figure S1 Concentration gradient binding validation for L-malate and succinate. DSF analysis was performed using ligand concentrations of 10 μM, 100 μM, 1 mM, 5 mM and 10 mM with LBD_{0646}. Data for (left) L-malate and (right) succinate are shown. ΔT_m values were shown for each compound tested relative to the T_m of the ligand-free protein

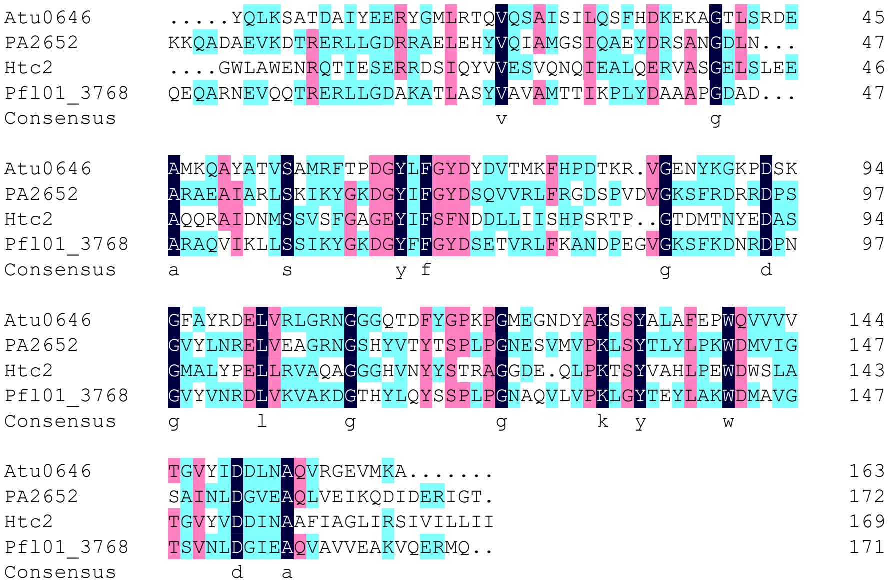

Figure S2 Sequence alignment of 4 sCache_2-type LBDs from C4-dicarboxylic acid chemoreceptors. The color bar above the sequences indicated the conservation level, with dark blue background representing identical residues

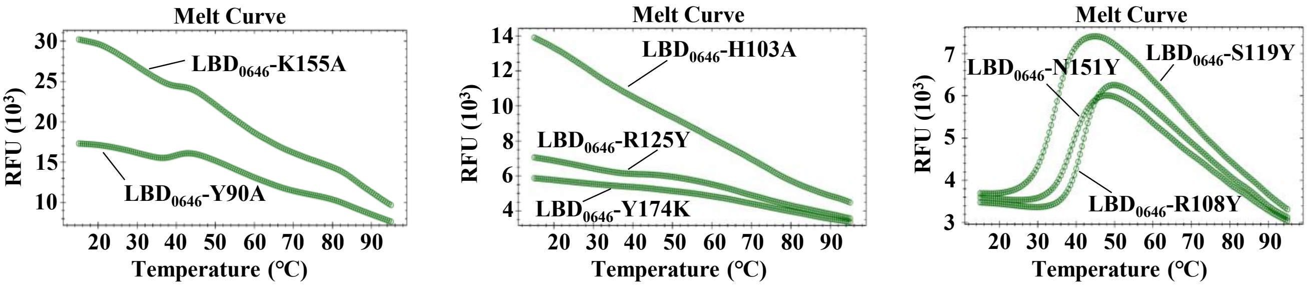

Figure S3 Thermal denaturation curves of 8 LBD_{0646} variants. Eight LBD_{0646} variants (LBD_{0646}-Y90A, LBD_{0646}-H103A, LBD_{0646}-K155A, LBD_{0646}-R108Y, LBD_{0646}-S119Y, $LBD0_{646}$-R125Y, LBD_{0646}-N151Y, and LBD_{0646}-Y174K) were expressed as N-terminal 6×His-tagged recombinant proteins and purified using a Ni-resin column. Thermal denaturation

of the purified LBD_{0646} variants was monitored by DSF and the resulting curves were used to assess their structural stability. Only three variants (LBD_{0646}-R108Y, LBD_{0646}-S119Y, and LBD_{0646}-N151Y) possess normal denaturation curves

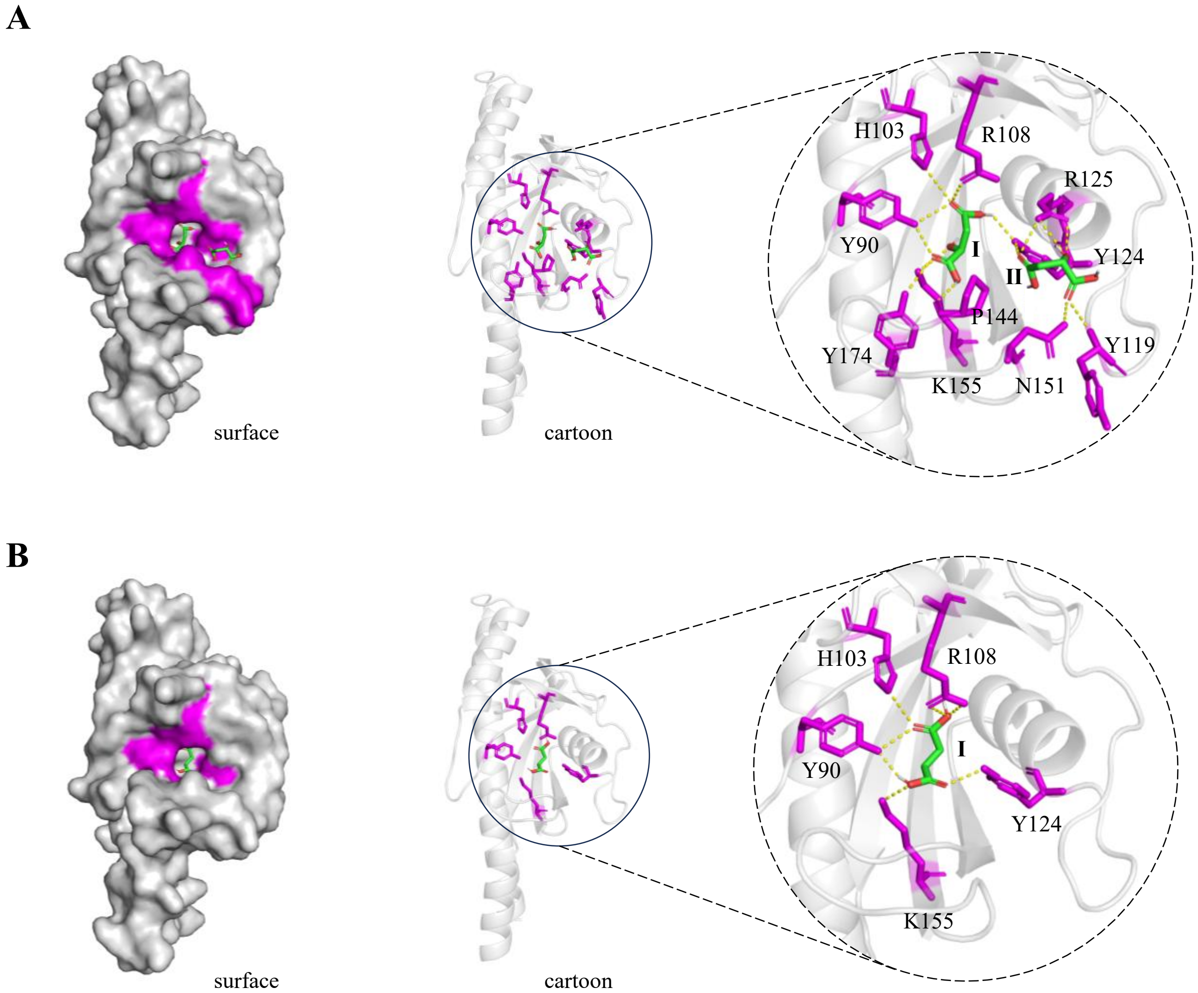

Figure S4 Ligand-binding models of LBD_{0646}-S119Y. The structural model of LBD_{0646}-S119Y was built by AlphaFold and molecular docking of L-malate (**A**) and succinate (**B**) to LBD_{0646}-S119Y was performed using AutoDock Vina. Modeling results are presented in surface and cartoon representations. The ligand-binding sites are zoomed in in the rightmost panels to show two binding modes (I and II). Ligands are shown as green sticks. Magenta regions (or sticks) indicate key residues involved in ligand binding. In this ligand-binding model of LBD0646-S119Y, the binding mode II of L-malate is altered by S119Y substitution, with its binding energy changing from -4.7 kcal/mol to -3.9 kcal/mol. The binding mode II of succinate is abolished by S119Y substitution

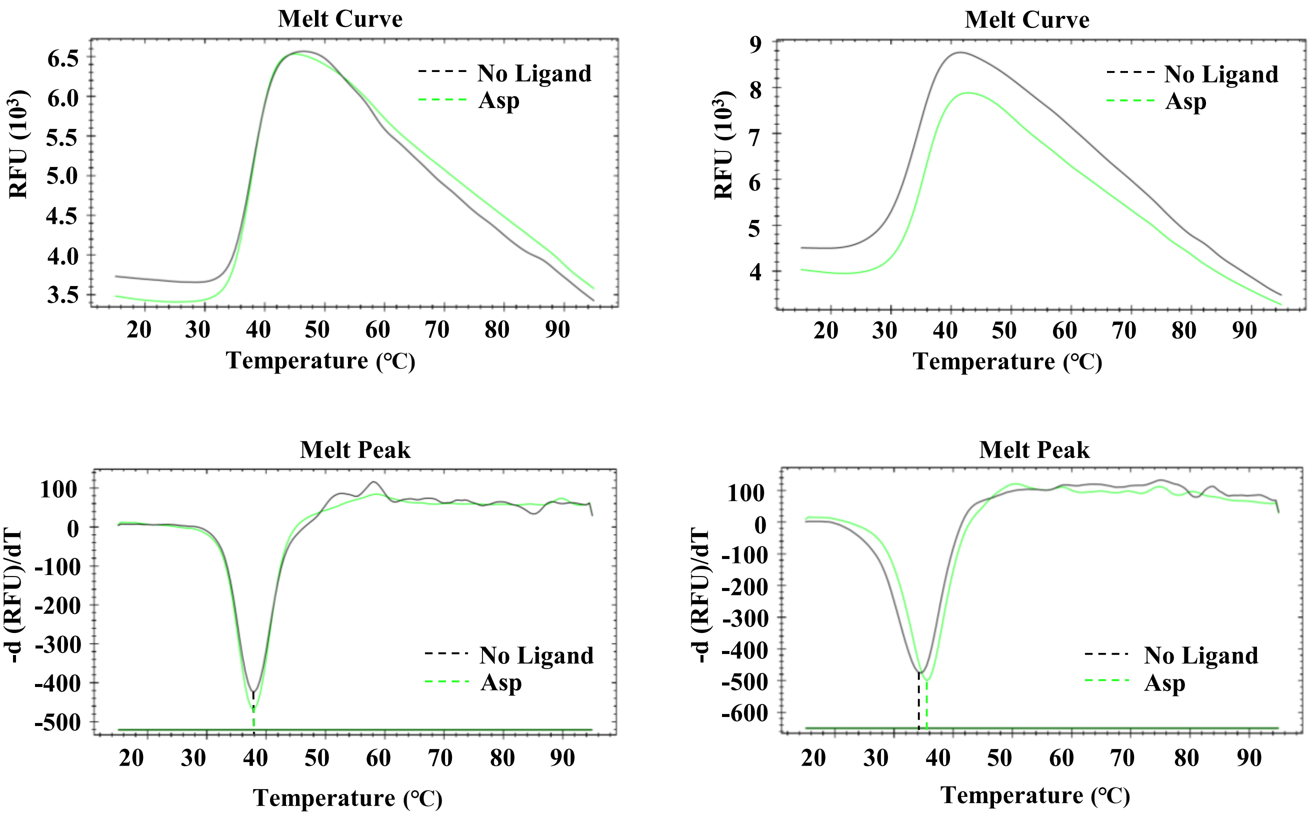

Figure S5 DSF determination of the binding of L-aspartate to the LBD_{0646} (left) and LBD_{0646}-S119Y variant (right). The upper panels show the thermal denaturation curves with or without Asp. The lower panel show the negative first derivative of the corresponding denaturation curves. The negative peaks indicate the protein melting temperature (T_m). The black dashed line and the green dashed line correspond to the absence and presence of L-aspartic acid

References

38. Guo, M.; Hou, Q.; Hew, C.L.; Pan, S.Q. Agrobacterium VirD2-binding protein is involved in tumorigenesis and redundantly encoded in conjugative transfer gene clusters. *Mol. Plant-Microbe Interact.* **2007**, *20*: 1201–1212.

62. Studier, F.W.; Moffatt, B.A. Use of bacteriophage T7 RNA polymerase to direct selective high-level expression of cloned genes. *J. Mol. Biol.* **1986**, *189*: 113–130.

63. Thomashow, M.F.; Nutter, R.; Montoya, A.L.; Gordon, M.P.; Nester, E.W. Integration and organization of Ti plasmid sequences in crown gall tumors. *Cell* **1980**, *19*: 729–739.

64. Hoang, T.T.; Karkhoff-Schweizer, R.R.; Kutchma, A.J.; Schweizer, H.P. A broad-host-range Flp-FRT recombination system for site-specific excision of chromosomally located DNA sequences: application for isolation of unmarked *Pseudomonas aeruginosa* mutants. *Gene* **1998**, *212*: 77–86.

65. Xiang, C.;Han, P.; Lutziger, I.; Wang, K.; Oliver, D.J. A mini binary vector series for plant transformation. *Plant Mol. Biol.* **1999**, *40*: 711–717.
